# Effects of a healthcare students’ prevention intervention for school children on their own substance use: a before-after study

**DOI:** 10.1186/s12909-023-04813-0

**Published:** 2023-11-07

**Authors:** Bastien Boussat, Mélanie Gaillet, Joey Fournier, Alizé Guyomard, Patrice François, Rebecca Shankland

**Affiliations:** 1 Service d’épidémiologie Et Évaluation Médicale, CHU Grenoble-Alpes, Boulevard de La Chantourne, 38700 La Tronche, France; 2 Laboratoire TIMC-IMAG, UMR 5525 CNRS, Université Grenoble Alpes, Grenoble, France; 3 Laboratoire DIPHE, Université Lumière Lyon 2, Lyon, France; 4 Institut Universitaire de France, Paris, France

**Keywords:** Student-led prevention, Psychosocial competences, Substance abuse, Healthcare students, Cognitive dissonance

## Abstract

**Background:**

Cognitive dissonance theory and research has suggested that engaging in prevention interventions for other students may be a means of reducing one’s own problematic behaviors in order to reduce potential cognitive dissonance. This study assessed the effects of a new mandatory prevention intervention program for healthcare students in France. The aim was to measure the effects of engaging in a prevention program in schools on the usual increase in substance use in student populations.

**Methods:**

Healthcare students were trained in a French university to develop psychosocial competences as a health promotion means (FEPS training) or more specifically to prevent substance use in teenagers (Unplugged program training). The students (*n* = 314) who accepted to take part in the study from both groups completed questionnaires before their interventions in schools, and at the end of the year, measuring their representations and behaviors regarding psychoactive substances.

**Results:**

The results indicated a significant reduction in alcohol consumption in terms of quantity, but no significant reduction in tobacco and marijuana consumption.

**Conclusions:**

This study showed that, contrary to the usual increase in substance use in students as they advance in their year, the students who took part in this study showed reduced self-reported consumption of alcohol after they had performed the prevention intervention in schools regardless of the type of training they had received (general health promotion vs. specific substance use prevention program). Limitations and future perspectives are discussed.

**Supplementary Information:**

The online version contains supplementary material available at 10.1186/s12909-023-04813-0.

## Background

### Vulnerabilities in student populations

Psychoactive substance abuse prevention is essential in student populations as increased consumption has been shown after integrating higher education [1]. Indeed, university students are more vulnerable to the onset of common mental health problems and psychological distress compared to the age-matched general population [2], which can increase substance abuse. The transition to higher education brings a host of new stressors such as leaving home, developing autonomy in daily tasks, reduced social support due to the new environment with new relationships to build, and academic pressure as well as decision-making challenges [3]. University students report loneliness and struggling to manage their time effectively because of a heavy curriculum [4]. These various stressors not only affect students’ normal day-to-day activities but also lead to sleep disorders, substance abuse and suicidal ideations [3]. In the student population, healthcare students are among the most subject to stress and burnout [5–8]. Clinical training is a significant component of students’ professional development as healthcare practitioners, but can also be highly stressful [9–11].

In order to reduce health risks and academic problems, a number of psychoactive substance abuse prevention programs for students have been implemented. However, research has underlined low motivation to attend substance abuse prevention programs [1].

### The sanitary service program

In 2018, French authorities launched a new mandatory action-training program in the initial training curricula of health professions [12–14]. This program, called “Sanitary Service”, aims to strengthen the acquisition of knowledge and skills in health promotion by students in health courses and to carry out prevention interventions with target populations, mainly middle and high school students. The objectives of the sanitary service are also to learn to work in an inter-professional manner and to know how to manage a project [15–17]. The program lasts a total of 3 months and includes a training period for the students, an intervention period with the public and an evaluation period.

The training institutes for medical doctors, pharmacists, physiotherapists and midwives in the Grenoble region have joined together in a health promotion program based on the European Unplugged program [18]. The Unplugged program aims to prevent addictive behaviors by developing psycho-social skills in schoolchildren and has shown a protective effect on the use of psychoactive substances [19, 20].

Thus, healthcare students are asked to engage in prevention programs in schools. This may at the same time become a means of increasing health promotion in these students by engaging them in the development and implementation of prevention programs. Indeed, research has shown that engaging youth in developing and facilitating prevention programs increases their own health behaviors [21–25].

### Cognitive dissonance theory

According to Cognitive Dissonance Theory [26], individuals seek consistency in their perceptions, thus when an inconsistency arises between attitudes and behaviors (i.e., dissonance), individuals tend to change their behavior to reduce internal discomfort. For example, middle school student smokers who created anti-smoking videos reduced their tobacco consumption [27].

Implementing a prevention program in schools can be considered as a Dissonance-Based Intervention which could allow for shifts from pro-psychoactive substance use norms (i.e. considered as a means to be more socially integrated at university), to psychoactive substance-averse norms, which could then result in less substance use. Past research has shown that Dissonance-Based Interventions increase positive behavioral change with a variety of disorders including substance abuse [28]. Furthermore, considering healthcare students as prevention experts is imbedded in a strengths-based perspective rather than a problem-focused approach to their health behaviors [29], which may enhance their self-efficacy (perceived ability to perform a behavior; [30]) regarding health behaviors, thereby increasing health behaviors [31]. In addition, taking part in the development and implementation of prevention programs increases the sense of ownership which has been identified as a critical aspect of intervention efficacy [32]. These beneficial effects are also in line with cognitive sciences and education research literature which show that being in the position of helping others learn is a more powerful mechanism to gain awareness and insight than receiving information didactically [33].

The aim of the present study was to assess the effects of healthcare student-led prevention programs in schools on their own psychoactive substance representations and consumption. We hypothesized that facilitating prevention programs would significantly reduce healthcare students’ psychoactive substance consumption.

## Method

### Design

This was a self-administered questionnaire survey of healthcare students who participated in the Sanitary Service in the Grenoble region in France during their training. The same questionnaire was administered before and after the completion of the sanitary service. The questionnaire asked the respondents to create a code to protect their anonymity. The students had to create the same code on both questionnaires, allowing to preserve their anonymity and to link the 2 questionnaires of the same student. Participants signed an informed consent to take part in the study.

### Participants

The implementation of the 2018–2019 sanitary service in the Grenoble region involved 400 students: 207 students in third year of medicine, 93 students in fifth year of pharmacy, 61 students in fourth year of physiotherapy and 39 students in second year of midwifery [34]. In December 2018, all students were invited to complete a first time the questionnaire during an information session (prior to the completion of sanitary service). It was submitted a second time in May 2019 during courses or exams (approximately 1 month after the completion of the program). The questionnaires were filed by students using pen-and-paper.

### The sanitary service

The health care students in medicine, pharmacy, physical therapy, midwifery and nursing were enrolled in a common program including specific training in health promotion and primary prevention interventions. Sanitary service was integrated into the 2nd year of midwifery, the 3rd year of medicine, the 4th year of physiotherapy and 5th year of pharmacy, following the ministerial decree specifying the Sanitary Service outline. The training of the students included two components: the provision of basic knowledge and the acquisition of educational skills. Core knowledge was provided through a digital platform with lectures and self-tests divided into seven modules. Two general knowledge modules introduced health education and the role of the school in health promotion. The other five modules focused on prevention topics: addictions and addictive behaviors, healthy living (diet and physical activity), sexual health, mental health and vaccinations. This training package corresponded to 30 h of instruction per student.

The educational skills were provided through a two-day seminar in small groups of 22 to 28 students, i.e., 16 h of instruction per student. The focus was on learning how to construct a health education intervention and apply group facilitation techniques. These seminars were based on two kinds of programs: the Unplugged program or on a Health Education and Promotion Training (HEPT) program. The Unplugged program is an evidence-based substance abuse prevention program designed for young people. It focuses on the development of psycho-social skills, on promoting healthy behaviors and preventing substance use, including alcohol, tobacco, and drugs. The program is typically delivered in school settings and aims to equip students with the knowledge and skills they need to make informed, healthy choices regarding substance use. Key features of the Unplugged program include interactive and engaging activities, peer-led discussions, and the involvement of parents and teachers in the prevention process. It emphasizes building protective factors and resilience in young individuals, which can reduce their vulnerability to substance abuse.

The HEPT program is a comprehensive and proactive approach with a broader focus. It aims to elicit representations related to prevention and health determinants, work on adopting an effective educational approach, familiarize participants with tools for designing and conducting prevention sessions in a school environment, understand group dynamics, and know how to evaluate one's actions. This program takes a holistic approach to health education, equipping participants with valuable skills and knowledge to promote health and well-being, extending beyond substance abuse prevention.

### Prevention interventions

The intervention sites were middle school and high schools, recruited by the rectorate from among respondents to a call for volunteers. The schools were invited to choose one or more prevention themes according to their needs and their own health education policy. The Unplugged program was offered to schools where one or more teachers had been trained in this program. For the other schools, the students built their intervention based on the HEPT program techniques. Students were divided into groups of 2 to 6, at least two different training fields to meet the requirement of multi-professionalism.

**Fig. 1:**
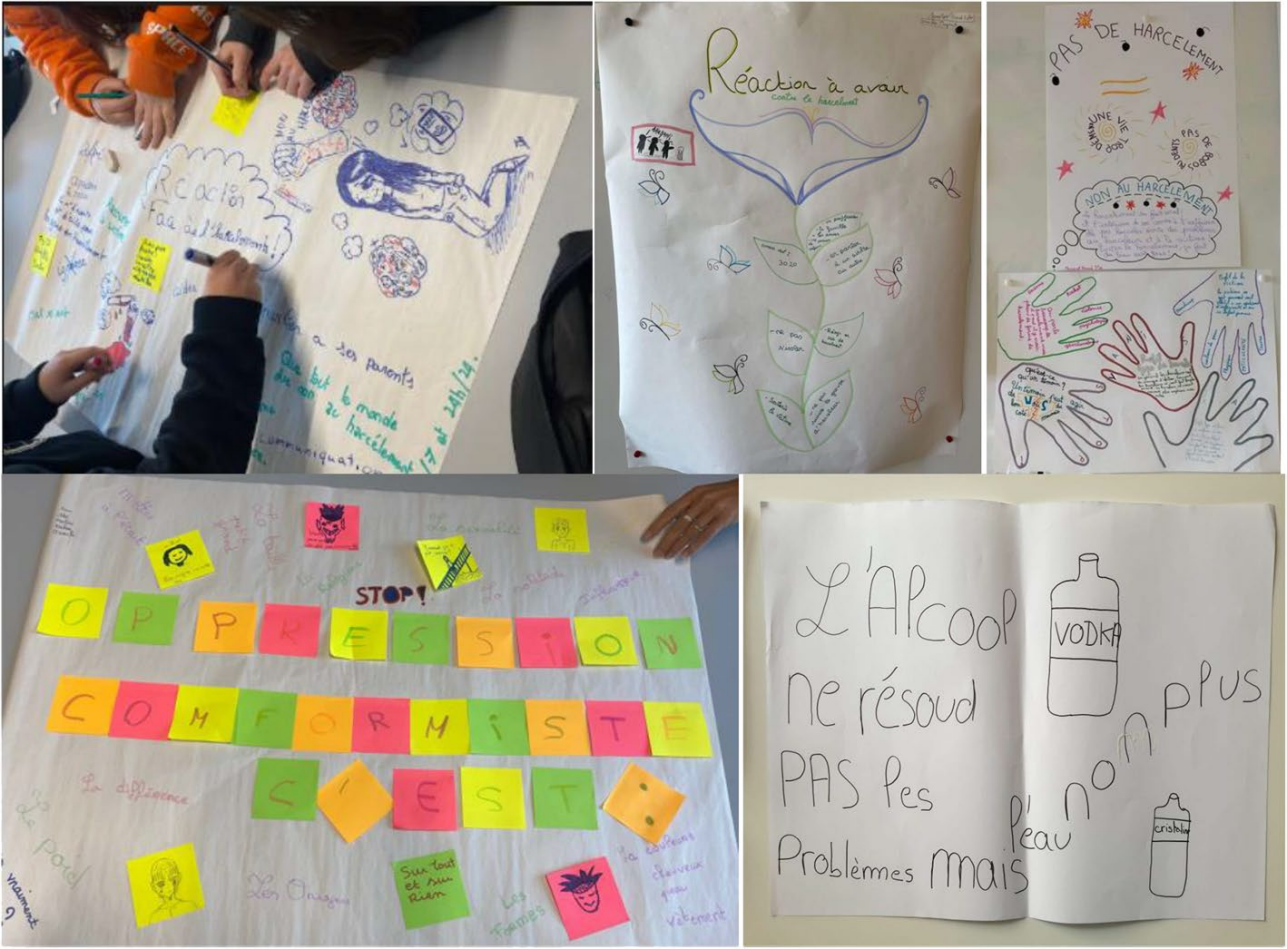
Photos taken in classrooms during the interventions (Unplugged and HEPT)

The details of the interventions carried out in the school establishments have been published elsewhere [34]. In summary, students were divided into 92 groups and conducted interventions in 91 establishments. Students were most often in groups of six. These groups were almost always multi-professional (96% of students). The host establishments were predominantly secondary education institutions (90 out of 91), including 60 middle schools and 30 high schools. The prevention interventions reached 7,926 individuals. The highest proportion of students was in the 5th grade (33.8%). The next most common grade levels were 10th grade (18.8%) and 6th grade (14.4%). The topics addressed by the students were diverse and sometimes multiple within the same student group (one to seven topics covered per group). In 55 establishments, students implemented the Unplugged program. In other establishments, students created programs tailored to requested themes using the tools taught in the HEPT training, with the most frequent themes being screens in 17 establishments and addictions in 12 establishments. The number of sessions conducted in a class by the student group was typically five sessions for Unplugged and four sessions for HEPT. Figure 1 displays a selection of visual posters created by children from several classes who participated in the Sanitary Service.

### Measures

The questionnaire was developed by researchers in social psychology and in epidemiology and prevention based on existing scales but selecting a small number of items or single items in order to keep the questionnaire as brief as possible. It included items from the Blitz questionnaire used by the Observatoire Territorial des Conduites à Risque de l’Adolescent (OTCRA) for an annual survey of middle school students. Two demographic items were used (age and gender). Three single items measured negative affect (depression, anxiety and irritability) on a 5-point scale ranging from 0 (never) to 4 (very often). Tobacco, alcohol and cannabis consumption were measured through frequency and quantity ordinal scales (the detailed scales were shown in the supplementary material 1). Finally, seven items related to alcohol consumption positive expectancy based on the French version [35] of the alcohol expectancy questionnaire [36], rated on a 5-point Likert scale ranging from 0 (totally disagree) to 4 (totally agree).

### Statistical analyses

Responses from each participant were matched with an anonymity code and entered into Excel. Ordinal responses were re-coded on a numerical scale from 0 to 4. Descriptive statistics, including means or medians for continuous variables and numbers and percentages for categorical variables, were used for reporting.

The normality of differences before and after was assessed for continuous variables through both graphical examination and the Shapiro–Wilk test. Comparisons between before and after responses were analyzed using paired t-tests or the Wilcoxon signed-rank test when appropriate for continuous outcomes, and McNemar’s test for categorical outcomes.

To study potential factors independently associated with self-reported alcohol consumption while accounting for the structure of repeated data and intra-individual correlation among participants (the same individuals responding before and after), we selected Generalized Linear Mixed Models (GLMM).

A significance level of 2-sided *P* < 0.05 was considered statistically significant. All statistical analyses were conducted using Stata Standard Edition version 16 (StataCorp).

## Results

### Sample characteristics

Of the 400 students participating in the Grenoble area sanitary service, 351 responded to the questionnaire before the program began and 367 after the program ended. The anonymity code generated by the students allowed us to match 314 pairs of questionnaires for the analyses. The final sample include 314 students (i.e. 79% of the students.), 158 in medicine, 67 in pharmacy, 52 in physiotherapy and 37 in midwifery. The mean age of the students was 21.9 years (*SD* = 2.3), and 229 (73%) were female (Table 1). The health education intervention was based on the Unplugged program for 184 students, on the HEPT program for 119 and unknown for 11.

**Table 1:** Characteristics of the students who answered T1 and T2 questionnaires (*n* = 314)

	N (%)
Age^a^, *mean* ± *SD*	21.6 ± 2.5
Female gender	231 (74)
Field of study	
Medicine	158 (50)
Pharmacy	67 (21)
Physiotherapy	52 (17)
Midwifery	37 (12)
Training program	
Unplugged training	184 (59)
HEPT training	119 (38)
No program specified	11 (4)

^a^Age: 4 missing data

### Change in negative affect and substance consumption

In terms of negative affect, there was a significant decrease in the scores of irritability (1.73 versus 1.88, *p* = 0.002) after the health service (Table 2).

**Table 2:** Comparison of scores before and after completion of the sanitary service^a^

Item	Before		After		p
	Mean	(SD)	Mean	(SD)	
Negative Affect					
Depression	1.40	(1.18)	1.30	(1.08)	0.10
Irritability	1.88	(1.06)	1.73	(1.07)	0.002
Anxiety	2.29	(1.21)	2.18	(1.07)	0.11
Tobacco use					
Frequency of use^b^	0.40	(0.88)	0.40	(0.83)	0.66
Number of cigarettes^b^	0.50	(1.10)	0.46	(1.03)	0.36
Alcohol consumption					
Frequency of consumption^b^	1.87	(0.93)	1.78	(0.94)	0.05
Number of glasses of drinks on a “drinking day”^b^	1.29	(1.27)	0.93	(0.99)	< 0.001
Cannabis consumption n (%)	81 (26)		73 (23)		0.19
Consumption before midday^b^	0.11	(0.41)	0.09	(0.35)	0.18
Consumption alone^b^	0.09	(0.50)	0.07	(0.37)	0.56

^a^Ranges (Min; Max) were (0;3) for Frequency of tobacco use

Ranges (Min; Max) were (0;4) for Depression, Irritability, Anxiety, Frequency of Alcohol consumption, Cannabis consumption before midday & alone

Ranges (Min; Max) were (0;5) for Number of glasses of drinks on a “drinking day”

Ranges (Min; Max) were (0;6) for Number of cigarettes

^b^Given the non-normality of the paired differences, the before after difference was statistically checked using Wilcoxon matched-pairs signed-rank tes

Regarding the consumption of psychoactive substances, there was a significant decrease in alcohol consumption in the quantity consumed (0.93 versus 1.29, *p* < 0.001). Tobacco consumption was initially quite low and the decrease in the daily number of cigarettes was not significant (0.46 versus 0.50, *p* = 0.36). The same was true for cannabis use, which was initially quite low and did not decrease significantly in either morning or solo use. The detailed results were shown in the supplementary material 1.

### Change in representations of alcohol consumption

The analysis of the questions concerning the representations of alcohol consumption showed significant changes in the students' statements after the health service (Table 3). There was a significant decrease in the scores of several positive propositions for alcohol consumption: "alcohol improves sexuality" (1.29 versus 1.59, *p* < 0.001), "with alcohol one feels stronger" (2.91 versus 3.02, *p* = 0.02) and "alcohol decreases tension" (2.31 versus 2.46, *p* = 0.01). However, the score of another positive proposition increased: "alcohol helps to think better" (0.71 versus 0.59, *p* = 0.01). And the score of a negative proposition decreased: "alcohol degrades the way people think" (3.13 versus 3.25, *p* = 0.02).

**Table 3:** Students' perceptions of alcohol use^a^

Question	Before		After		p
	Mean	(SD)	Mean	(SD)	
Alcohol generally has powerful positive effects on people (they feel good or happy, the future looks brighter)	1.97	(1.06)	1.95	(1.07)	0.69
Alcohol can help or hurt the way people get along (it helps people have a good time together; it makes people bad with others)	3.00	(0.77)	2.98	(0.74)	0.77
Alcohol helps people think better and makes it easier to coordinate (people understand things better, manage to do things better)	0.59	(0.66)	0.71	(0.80)	0.01
Alcohol improves sexuality (more pleasurable, feels more romantic or sexually aroused; makes sex easier)	1.59	(1.10)	1.29	(1.04)	< 0.001
Alcohol degrades the way people think and disrupts their coordination (they rush into things, act stupidly, have hangovers)	3.25	(0.71)	3.13	(0.79)	0.02
Alcohol makes people feel stronger and more powerful (easier to fight, speak in public, assert themselves in front of others)	3.02	(0.75)	2.91	(0.75)	0.02
Alcohol helps the person relax, feel less tense, and not dwell on mistakes made at school or work	2.46	(0.94)	2.31	(0.90)	0.01

^a^Ranges (Min; Max) were (0;4)

In multivariate analysis, there were no characteristics independently associated with the observed decrease in alcohol consumption (Table 4).

**Table 4:** Multivariate analysis to explore potential association with alcohol consumption^a^ (*n* = 314)

Characteristics	Alcohol consumption (Number of drinks)						
	Before		After		p	Coefficient	[95% CI]
	Mean	(SD)	Mean	(SD)			
Gender							
Men	2.54	(1.60)	2.00	(1.23)	reference		
Women	2.11	(1.27)	1.80	(1.06)	0.19	-0.15	[-0.39; 0.08]
Training program							
Unplugged	2.28	(1.40)	1.92	(1.11)	reference		
HEPT	2.10	(1.34)	1.71	(1.04)	0.62	0.05	[-0.15; 0.38]
Faculty							
Medicine	2.37	(1.42)	1.99	(1.14)	reference		
Pharmacy	1.88	(1.35)	1.55	(0.93)	0.33	0.13	[-0.13; 0.38]
Physical therapy	2.02	(0.96)	1.67	(0.94)	0.49	0.10	[-0.18; 0.38]
Midwifery	2.47	(1.61)	2.11	(1.33)	0.67	0.07	[-0.25; 0.39]

^a^GLMM model where identity was carried as random-effect and Gender, training Program and Faculty were carried as fixed-effects

## Discussion

The aim of this study was to assess the effects of a peer-led prevention training, termed “sanitary service”, for healthcare students on their mental health and substance use behaviors. The results highlighted that healthcare students’ participation in the sanitary service appeared to have a positive effect on their irritability, as well as on their alcohol consumption, and on their representations of the effects of alcohol consumption.

One of the explanations for the improvement in negative affect may be explained by the anxiety-provoking nature of the introduction of a new and mandatory peer-led prevention program which healthcare students are asked to develop in school contexts [37]. Indeed, a survey of students involved in sanitary service at another university showed that students had a negative perception of the sanitary service before the training and that this perception became positive afterwards [12]. After the completion of the sanitary service, students increased levels of self-esteem and changed their representations and behaviors regarding prevention practices [12, 38]. Therefore, these effects may reduce irritability in university healthcare students compared to before the training. Another explanation may be that as the training proposed in University Grenoble Alps is based on psychosocial development, the students may have developed their own psychosocial competences through the training they received, which in turn may have helped them face the daily challenges with a greater ability to cope (see the meta-analysis by Durlak et al. on the effects of psychosocial competences) [39].

Concerning alcohol consumption, it is known that it is a problem among young people and particularly among medical students [40–42]. More specifically, a festive consumption often in the form of binge-drinking is very present in the social life of students [42, 43]. Several studies have shown that reinforced training on prevention and health education can change their representations of alcohol abuse and even reduce their consumption [44]. This can be increased by the peer-led nature of the program in our study as the students were then asked to propose a program in school settings on the development of psychosocial competences and substance use prevention [45]. Indeed, research in the field of dissonance-based interventions has underlined the effects of peer-led prevention programs on their own levels of consumption as the message given to other students increases cognitive dissonance in themselves, which in turn encourages the reduction of their own consumption [28].

Although these results are promising, some limitations need to be underlined. The main limitation of the study is the absence of an inactive control condition which does not allow for the assertion that the cause of the observed changes is the participation in the sanitary service. In this study we compared two types of trainings: one that was only based on the development of psychosocial competences while the other program comprised specific content on substance use prevention. As no difference was shown between these two trainings, further research is needed in order to identify the mechanisms through which these interventions may be effective, and to compare the results with a wait-list control group who does not benefit from such training at the same period of the year. In line with these perspectives, future research may want to assess the development of psychosocial competences in healthcare students which could be one way of explaining the effectiveness of such trainings. Finally, the second wave of questionnaires was conducted only 1 month after the completion of our students' interventions. Therefore, we cannot determine whether the effects persist in the long term.

To conclude, this pilot study showed promising results on negative affect and alcohol consumption in healthcare students, but further studies are needed using a controlled design and measures of potential mechanisms of action.

### Supplementary Information

**Additional file 1:** **Supplemental file 1.** Expanded responses to the close questions of the questionnaire.

## Data Availability

The datasets generated and/or analysed during the current study are available from the corresponding author on reasonable request.
