# Supplementary material for: Effects of a healthcare students’ prevention intervention for school children on their own substance use: a before-after study
Source: BMC Med Educ. 2023 Nov 7;23:841. doi: 10.1186/s12909-023-04813-0 (PMC10631018; doi:10.1186/s12909-023-04813-0)
Supplement: Supplementary file 1 — Additional file 1: Supplemental file 1. Expanded responses to the close questions of the questionnaire. [file 12909_2023_4813_MOESM1_ESM.docx]

Supplemental File 1. Expanded responses to the close questions of the questionnaire

|  | Before | | After | |  |
| --- | --- | --- | --- | --- | --- |
| Negative Affect | N | (%) | N | (%) | P-value |
| Depression, mean (SD) | 1.40 | (1.18) | 1.30 | (1.08) | 0.10 |
| 0 = Never | 90 | (29) | 87 | (28) |  |
| 1 = Rarely | 82 | (26) | 105 | (33) |  |
| 2 = Occasionally | 84 | (27) | 70 | (22) |  |
| 3 = Quite Often | 42 | (13) | 46 | (15) |  |
| 4 = Very Often | 16 | (5) | 6 | (2) |  |
| Irritability, mean (SD) | 1.88 | (1.06) | 1.73 | (1.07) | 0.002 |
| 0 = Never | 34 | (11) | 42 | (13) |  |
| 1 = Rarely | 84 | (27) | 94 | (30) |  |
| 2 = Occasionally | 94 | (30) | 100 | (32) |  |
| 3 = Quite Often | 91 | (29) | 64 | (20) |  |
| 4 = Very Often | 11 | (4) | 14 | (4) |  |
| Anxiety, mean (SD) | 2.29 | (1.21) | 2.18 | (1.07) | 0.11 |
| 0 = Never | 29 | (9) | 15 | (5) |  |
| 1 = Rarely | 53 | (17) | 78 | (25) |  |
| 2 = Occasionally | 87 | (28) | 89 | (28) |  |
| 3 = Quite Often | 87 | (28) | 98 | (31) |  |
| 4 = Very Often | 58 | (18) | 34 | (11) |  |
| Tobacco use | N | (%) | N | (%) |  |
| Frequency of use*, mean (SD) | 0.40 | (0.88) | 0.40 | (0.83) | 0.66 |
| 0 = Does not smoke | 248 | (79) | 239 | (76) |  |
| 1 = < 1 time per week | 30 | (10) | 41 | (13) |  |
| 2 = ≥ 1 time per week | 12 | (4) | 16 | (5) |  |
| 3 = Every day | 24 | (8) | 18 | (6) |  |
| Number of cigarettes*, mean (SD) | 0.50 | (1.10) | 0.46 | (1.03) | 0.36 |
| 0 = No cigarettes | 242 | (77) | 246 | (78) |  |
| 1 = < 1 cigarette per week | 29 | (9) | 25 | (8) |  |
| 2 = < 1 cigarette per day | 14 | (4) | 22 | (7) |  |
| 3 = 1-5 cigarettes per day | 21 | (7) | 11 | (4) |  |
| 4 = 6-10 cigarettes per day | 4 | (1) | 8 | (3) |  |
| 5 = 11-20 cigarettes per day | 2 | (1) | 1 | (0) |  |
| 6 = >20 cigarettes per day | 2 | (1) | 1 | (0) |  |
| Alcohol consumption | N | (%) | N | (%) |  |
| Frequency of consumption*, mean (SD) | 1.87 | (0.93) | 1.78 | (0.94) | 0.02 |
| 0 = Never | 30 | (10) | 34 | (11) |  |
| 1 = Once a month or less | 62 | (20) | 74 | (24) |  |
| 2 = 2-4 times a month | 147 | (47) | 138 | (44) |  |
| 3 = 2-3 times a week | 69 | (22) | 63 | (20) |  |
| 4 = More than 4 times a week | 6 | (2) | 5 | (2) |  |
| Number of glasses of drinks on a “drinking day” , mean (SD) | 1.29 | (1.27) | 0.93 | (0.99) | <0.001 |
| 0 = No drinks | 104 | (33) | 129 | (41) |  |
| 1 = 1-2 drinks | 101 | (32) | 107 | (34) |  |
| 2 = 3-4 drinks | 56 | (18) | 54 | (17) |  |
| 3 = 5-6 drinks | 21 | (7) | 18 | (6) |  |
| 4 = 7-8 drinks | 32 | (10) | 6 | (2) |  |
| 5 = 10 drinks or more | 0 | (0) | 0 | (0) |  |
| Cannabis consumption | N | (%) | N | (%) |  |
| Consumption before midday*, mean (SD) | 0.11 | (0.41) | 0.09 | (0.35) | 0.18 |
| 0 = Never | 287 | (91) | 292 | (93) |  |
| 1 = Rarely | 23 | (7) | 18 | (6) |  |
| 2 = Occasionally | 2 | (1) | 3 | (1) |  |
| 3 = Quite Often | 1 | (0) | 1 | (0) |  |
| 4 = Very Often | 1 | (0) | 0 | (0) |  |
| Consumption alone*, mean (SD) | 0.09 | (0.50) | 0.07 | (0.37) | 0.56 |
| 0 = Never | 302 | (96) | 301 | (96) |  |
| 1 = Rarely | 4 | (1) | 6 | (2) |  |
| 2 = Occasionally | 1 | (0) | 5 | (2) |  |
| 3 = Quite Often | 6 | (2) | 2 | (1) |  |
| 4 = Very Often | 1 | (0) | 0 | (0) |  |
